# Morphological characteristics of the blackspot seabream (*Pagellus bogaraveo*) tongue: A structural and immunohistochemical study

**DOI:** 10.1111/ahe.12769

**Published:** 2021-11-24

**Authors:** Francesco Abbate, Maria Cristina Guerrera, Maria Levanti, Rosaria Laurà, Marialuisa Aragona, Kamel Mhalhel, Giuseppe Montalbano, Antonino Germanà

**Affiliations:** ^1^ Department of Veterinary Sciences University of Messina Messina Italy

**Keywords:** fish, immunohistochemistry, light microscopy, scanning electron microscopy, tongue

## Abstract

The blackspot seabream (*Pagellus bogaraveo*, Brünnich, 1768) is an omnivorous, predominantly carnivorous fish. In aquaculture, it is fed with pellets rich in proteins and fat. The morphological and functional aspects of the fish tongue, the feeding modality and the tasting capacity are strictly related. Therefore, the aim of this study was to describe by scanning electron, light and confocal laser microscopy, the morphological characteristics of the tongue in this species. It showed an apex, a body and a root. There were rows of teeth on the edges of the mouth and taste pores on all the tongue dorsal surface with folds and furrows. In addition, body and root showed several fungiform‐like papillae in the mucosa of the folds, covered by a weakly keratinized stratified squamous epithelium, can be observed. The papillae were innervated by S100 positive fibres. In the apex, a mesenchymal tissue with vimentin positive star‐shaped stem cells was evident. The results could give a support for a wider use of the blackspot seabream as a farmed species, considering the morphological data as correlated with the potentiality of food discrimination. This provides a basis for possible applications in feeding strategies. The presence, localization and characteristics of the mesenchymal stem cells, as seen also in previous studies, could represent a further basis for future applications in clinical trials.

## 1 INTRODUCTION

The blackspot seabream (*Pagellus bogaraveo*, Brünnich, 1768) is a bottom feeder fish of the family Sparidae Perciformes, Teleostei, widely present in the Eastern Atlantic and the western Mediterranean. It lives close to sandy and pebbly depths mixed with rocks and debris and on muddy‐arenous depths. It feeds on a wide variety of organisms, such as molluscs, crustaceans and small fish distributed along the entire water column. Currently, the species most frequently bred in Italy are the gilthead seabream (*Sparus aurata* Linnaeus), the European seabass (*Dicentrarchus labrax*, Linnaeus) and the rainbow trout (*Onchorinchus mykiss)*. In the last years, there is an increasing interest for new aquaculture species, through diversifying the aquaculture industry. The blackspot seabream is one of these possible alternatives to the commonly used fish for the high quality of its flesh and the high nutritive and commercial importance. The breeding of this species is already widely accepted in Spain, especially in the Azores. The blackspot seabream is also used to produce fish meal and oil. These are alternative and equivalent protein and lipid sources easily available, cheaper and with less environmental impact comparable to those of vegetable origin (Iaconisi et al., 2017; Micale et al., 2011). Therefore, this study aims to evaluate the morphology of the oral cavity, especially of the tongue, in relation to function. It has been shown that the capacity and modality of feeding are strictly related to the morphofunctional adaptations of fish to the environment, thus explaining the several differences observed in the presence, morphology, abundance and distribution of taste buds, papillae with mechanic or sensitive properties and teeth of different shapes in several species of fish (Abbate et al., 2006, 2017, 2020b, 2020a, 2012b, 2012a; Germanà et al., 2009; Amato et al., 2012; Dos Santos et al., 2015; Sadeghinezhad et al., 2015; Guerrera et al., 2015; Mahmoud et al., 2016; Kettratad et al., 2017; Levanti et al., 2017; Ikpegbu et al., 2019; Kasumyan, 2019). Also, a strict comparative correlations with numerous studies carried out in other vertebrates like birds and reptiles are significative (Abbate et al., 2020c, 2010, 2009, 2008; Erdoğan and Alan, 2012; Erdoğan and Iwasaki, 2014; Herrel et al., 2014; Cizek et al., 2019; Bels et al., 2020). In addition, recent data demonstrate that the role of some hormones is strictly related to the anatomical aspects of the digestive system and particularly of the teleosts oral cavity (Montalbano et al., 2016, 2018a; Mania et al., 2017; Audira et al., 2018; Carnovali et al., 2018). The relevance of this study is also demonstrated in previously investigated farmed fish (Abbate et al., 2020b, 2020a, 2012b, 2012a), showing that is strictly related to the correlations between the tongue morphology and the feeding habits. The adaptive changes in the morphology of fish oral cavity are in close relationship with the feeding function, with a significant influence on the processes of food intake and taste. This could help to identify new and better methods of farmed fish breeding, with regard to nutrition. It will help to improve the quality of the fish product. There are scant data on the anatomy of the oral cavity and tongue in Sparidae, and none to our knowledge exist for blackspot seabream. The aim of this investigation was to describe by scanning electron, light and confocal laser microscopy the characteristics of the tongue in blackspot seabream.

## 2 MATERIALS AND METHODS

The heads of 20 specimens (12 male and 8 female) of fresh blackspot seabreams of commercial size, (between 800 and 900 g) obtained from the fish markets of Messina, were used. The commercial size is typical of adult fish of this species that came from the aquaculture companies as certified by the seller. All these fish were intended for human consumption. After decapitation, better exposure of the tongue, the temporo‐mandibular joints were also disarticulated and cut. The tongues were dissected out and washed in 5% neutral Extran (Merck), which is a cleansing solution commonly used to remove the mucus.

### 2.1 Scanning electron microscopy

The samples of 10 fresh specimens (6 male and 4 female) were fixed in 2.5% glutaraldehyde in Sorensen phosphate buffer 0.1 M. After several rinsing in the same phosphate buffer, they were dehydrated in a graded alcohols series (50°, 70°, 80°, 95° and 100°, 1 hr for each step), critical point dried in a Balzers CPD 030 and then sputter‐coated with 3 nm gold in a Balzers BAL‐TEC SCD 050. Processed samples were examined under a Zeiss EVO LS 10 operating with an accelerating voltage of 20 kv (Modina et al., 2007).

### 2.2 Light microscopy

Tissue samples of 5 fresh specimens (3 male and 2 female) were fixed in Bouin fixative for 24 hr, dehydrated and routinely embedded in paraffin. As in previous studies on tongue (Abbate et al., 2006, 2017, 2020b, 2020a, 2012b, 2012a), about 10 µm thick transversal, horizontal and sagittal serial sections were obtained using Leica RM 2135 microtome (Leica microsistems Nussloch GmbH). Sections were mounted on microscope slides and stained with Masson's trichrome and with aniline blue 04‐010802 (Bio‐Optica). Weigert's iron haematoxylin for nuclei staining and aniline blue for connective tissue stain were used. After rinsing in distilled water, the sections were submitted to the reagents, as mentioned in the product datasheet, washed in distilled water and rapidly dehydrated through ascending alcohols, clarified in xylene and mounted using Eukitt mounting medium # ref. 09‐00250 (Bio‐Optica). Slides were observed under a Leica DMRB light microscope (Leica microsistems GmbH), and sections were captured with Leica camera MC 120 HD (Leica microsistems GmbH) (Garcia‐Suarez et al., 2018).

### 2.3 Laser confocal microscopy

The samples of 5 fresh specimens (3 male and 2 female) were fixed in Bouin's fixative for 24 hr and processed for routine paraffin embedding. The blocks were cut in 10 µm thick sections, as carried out for light microscopy. Serial transverse, horizontal and sagittal sections were mounted on gelatin‐coated microscope slides and processed for immunofluorescence. Following dehydration, rehydrated sections were washed with Tris–HCL (0.05 M, pH 7.5) containing 0.1% bovine serum albumin and 0.2% Triton X‐100. The endogenous peroxidase activity and non‐specific binding were blocked using 3% hydrogen peroxide and 50% foetal bovine serum. Thereafter, sections were incubated in a humid chamber overnight at 4°C with mouse anti‐vimentin (Serotec) No: 201100, dil. 1:100) and FLEX Polyclonal Rabbit Anti‐S100 Ready‐to‐Use (Dako Omnis) at dilution of 1:200. Subsequently, the sections were rinsed in PBS buffer. Then, they were incubated for 90 min at room temperature, respectively, with Alexa fluor 488‐ donkey anti‐mouse IgG (H+L) (Invitrogen, A 21202) at dilution of 1:300 and Alexa fluor 594 Goat anti‐rabbit IgG (H+L) Cross‐Adsorbed Secondary Antibody Catalog **#** A 11012 (Thermo Fisher).

For negative controls, representative sections were incubated with non‐immune rabbit sera instead of the primary antibodies, or omitting the primary antibodies, following the same procedure previously described (Viña et al., 2015). Under these conditions, no positive immunostaining was observed (data not shown).

The study was carried out on fresh fish obtained from common markets and intended for human consumption; therefore, no approval was necessary from local and/or national institutional animal use committee. However, all experimental processes followed the EU Directive 2010/63/EU in the use of animals for experimental purposes.

## 3 RESULTS

### 3.1 Gross results

**FIGURE 1:**
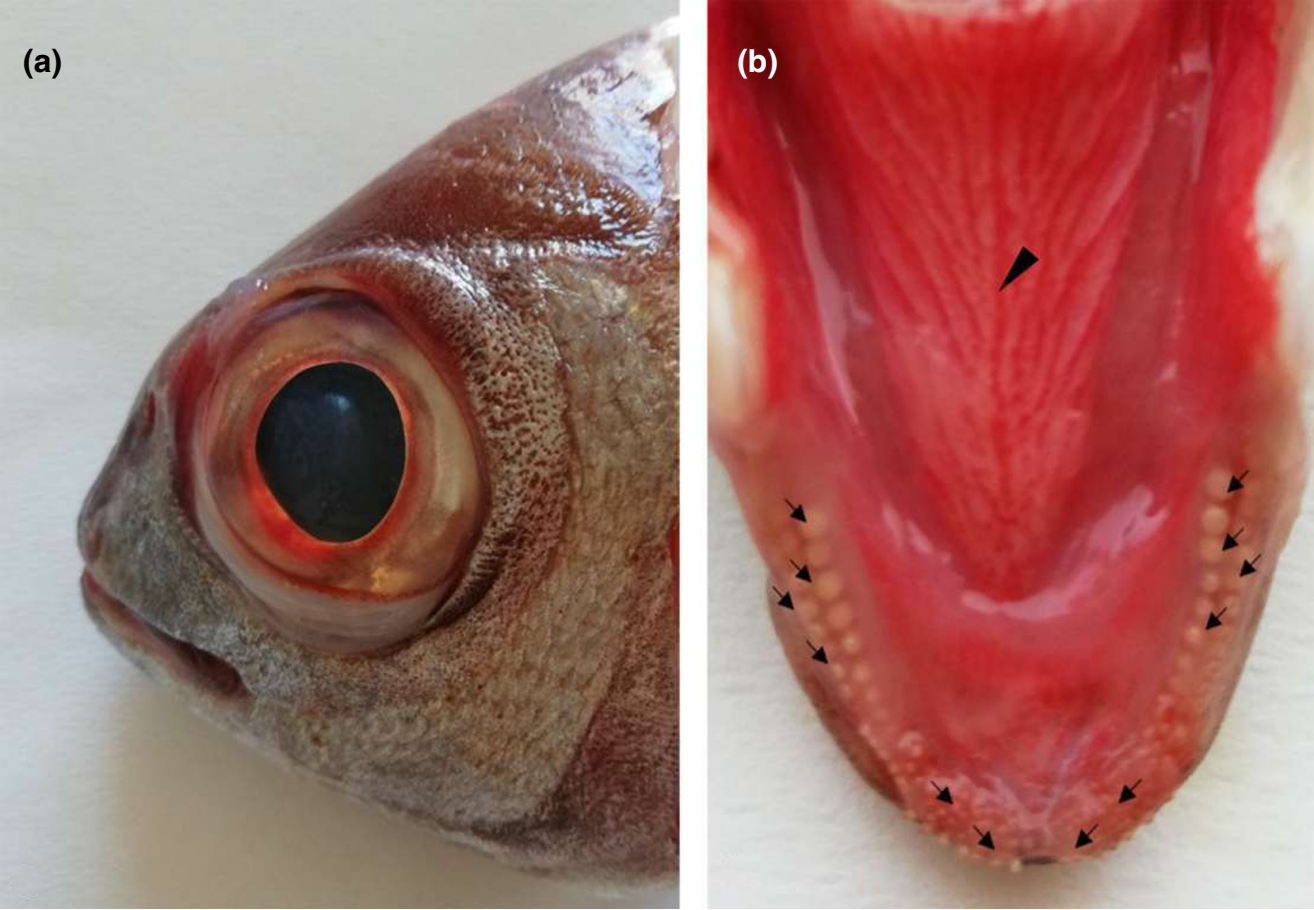
Gross photograph showing (a) macroscopical aspect of the blackspot seabream head and (b) the tongue dorsal surface: The arrows indicate teeth of various shapes, arranged in rows. The arrowhead indicates the median raphe

**FIGURE 2:**
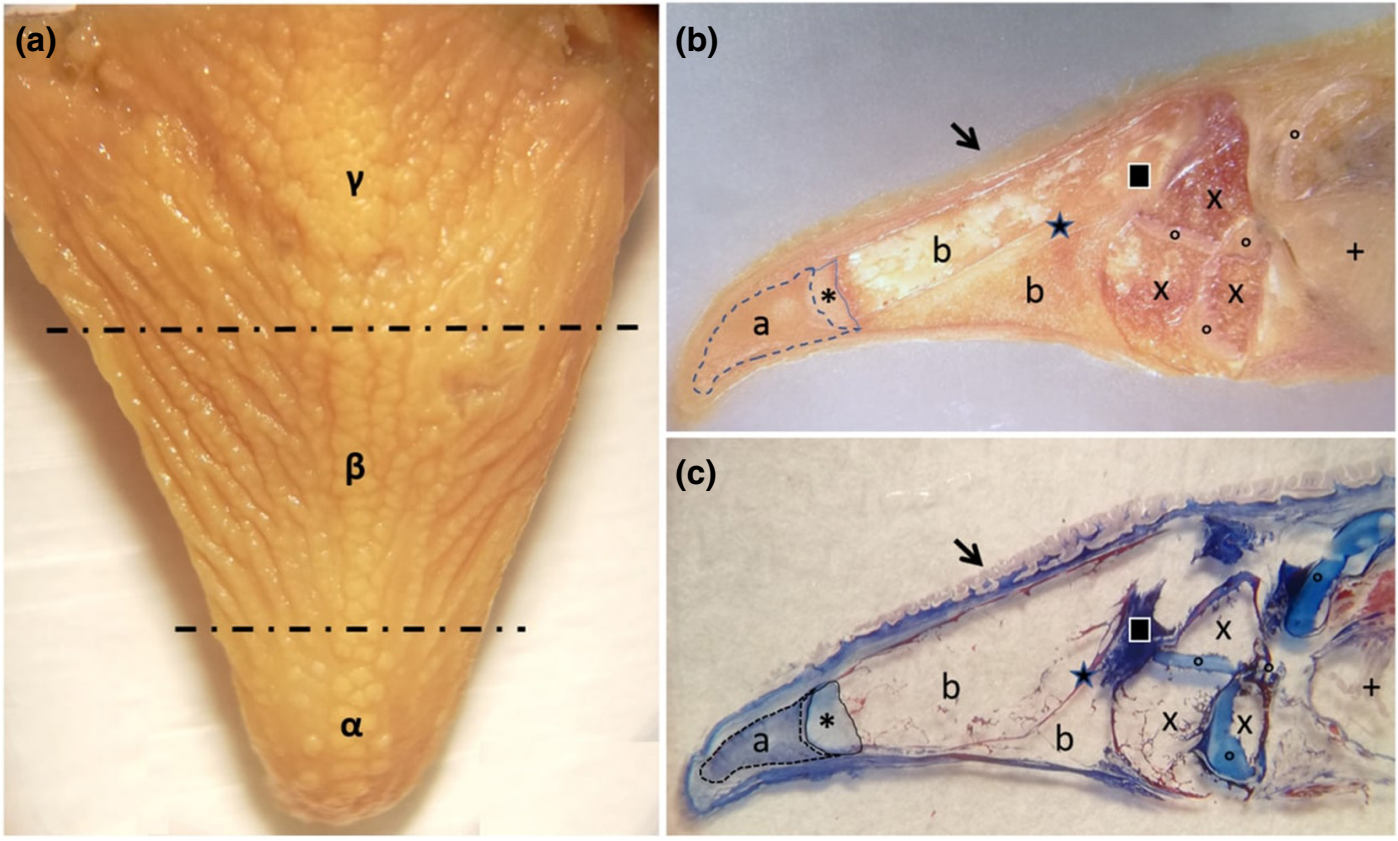
Gross photograph showing (a) macroscopical aspect of the blackspot seabream tongue, which is divided in three areas: the apex (α), the body (β) and the root (γ). Stereomicrographs (b and c) sagittal sections of the tongue: The arrow indicates the tongue dorsal surface covered by several papillae. At the apex level, a pad of mesenchimal tissue is evident (a), with aborally a cartilagineous tissue (asterisk). From the aboral portion of cartilagineous tissue, bone trabeculae were evident, originated by indirect ossification, delimitating niches of adipose tissue (b). An area of compact bone tissue (star) originates through a process of indirect ossification to replace a pre‐existing cartilagineous tissue (square). Adipose tissue (×), areas of hyaline cartilage (circles) and gills (+) characterize the aboral areas of the tongue stratigraphy

The snout was distinct with a shorter diameter than that of the eye (Figure 1a). The jaws (upper and lower) showed series of teeth of various shapes placed in the cranio‐lateral margins of the oral cavity floor (Figure 1b). The tongue had a pyramidal shape with an irregular dorsal surface which was characterized by several folds of the mucosa. These folds were apparent on both sides of the tongue. They showed a latero‐medial orientation and aboral inclination that converged on a median raphe (Figure 1b). An apex, a body and a root can be distinguished (Figure 2a). Several papillae were scattered on the dorsal surface of the tongue. A stratigraphy demonstrated different areas of the tongue. In the apex, a pad of mesenchymal and cartilaginous tissue, aborally placed, can be observed. Bone trabeculae delimitate niches of adipose tissue. An area of compact bone tissue replaces a pre‐existing cartilagineous tissue (Figure 2a–c).

### 3.2 Scanning electron microscopy

**FIGURE 3:**
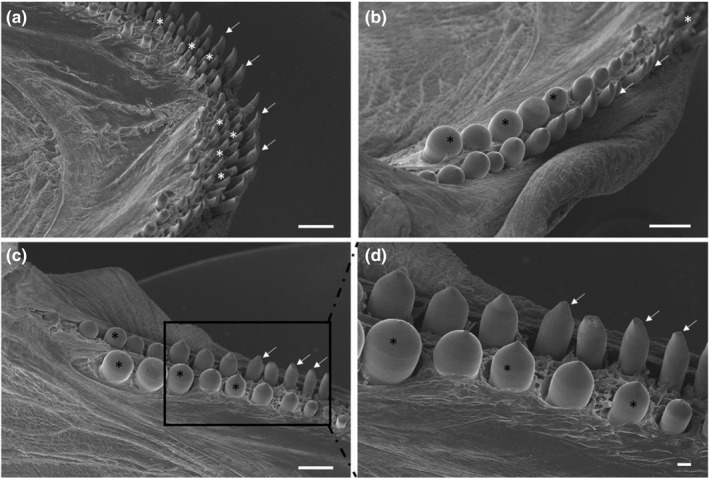
(a) Scanning electron micrograph of the rostral part of the oral cavity with cardiform (white asterisks) and caniniform teeth with aborally curved tips (arrows). (b and c) The molariphorm (black asterisks), caniniform (arrows) and cardiform (white asterisk) teeth on the oral cavity margins. (d) At higher magnification, the molariform (asterisks) and caniniform (arrows) teeth. Scale bar: a, b, c: 1mm; d: 200 μm

**FIGURE 4:**
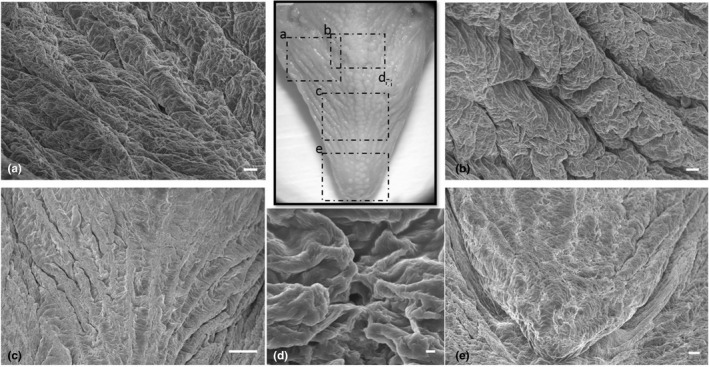
(a–c,). Scanning electron micrograph of the irregular dorsal surface of the tongue with prominent folds and deep furrows, with a latero‐medial orientation fading towards the apex. (d) Taste pores were observed among the folds. (e) The dorsal surface of the apex with less prominent folds. Scale bar: a, b: 100 μm; c: 1mm; d: 10 μm; e: 200 μm

An external row of pointed conical teeth was present in the oral cavity floor, with a slightly curved apex, an oro‐aboral orientation and an innermost series of slightly smaller cardiform teeth. Cardiform teeth are pointed, thin and short teeth, often numerous, present in many fishes that have multiple rowed teeth as American catfish (Ictaluridae), perches (Percidae) and many sea basses (Serranidae). They are followed, posteriorly, by two‐three rows of molariform teeth (Figure 3a–d). The dorsal surface of the tongue appeared irregular due to the presence of prominent folds and deep furrows along the entire extension that disappeared towards the apex (Figure 4a–d). No significant differences in the morphological aspects of the folds were observed in the different areas (apex, body and root) of the tongue. The folds were characterized by a very irregular surface, but all were latero‐medially oriented and converged from the lateral margins of the tongue towards a median raphe. Taste pores, with a sensory function, are scattered along the entire dorsal surface and are, in most cases, observed among the folds (Figure 4d). No taste buds, searched throughout the tongue, were present on the surface.

### 3.3 Light and confocal laser microscopy

**FIGURE 5:**
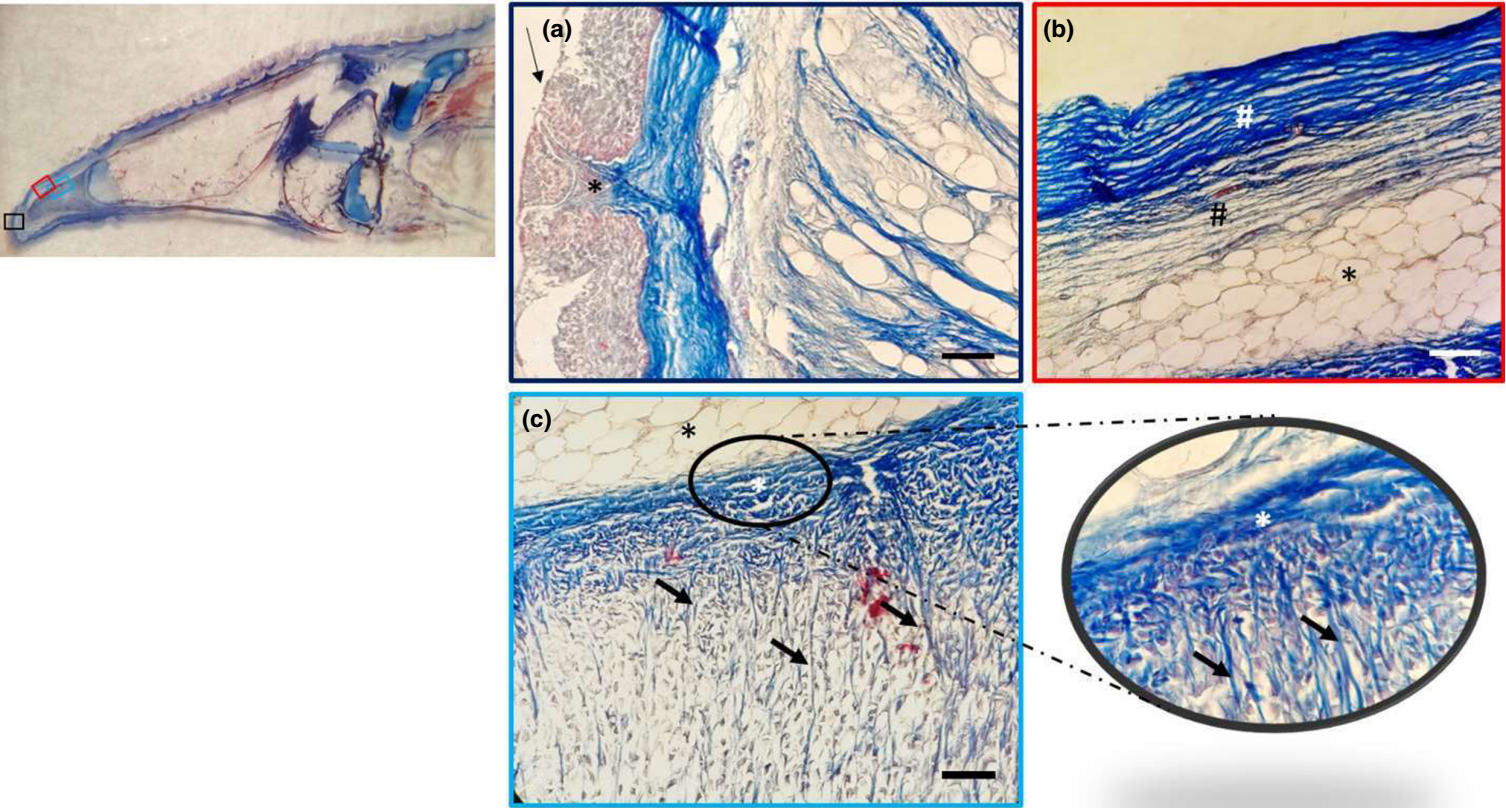
Light micrographs (Masson's trichrome with aniline blue staining): (a) In the apex, a weakly keratinized stratified squamous epithelium (arrow) and connective papillae (asterisk), showing between the epithelial laminae. (b) The dense fibrillar connective tissue of the papillae with abundant collagen fibres (white hashtag) and the deeper loose fibrillar connective tissue (black hashtag), which continued with a pad of unilocular adipose tissue (asterisk). (c) Unilocular adipose tissue (black asterisk); the dense connective tissue (white asterisk) forms septa (arrows) outlining niches of cells, at higher magnification in the insert. Scale bar: 20 μm

**FIGURE 6:**
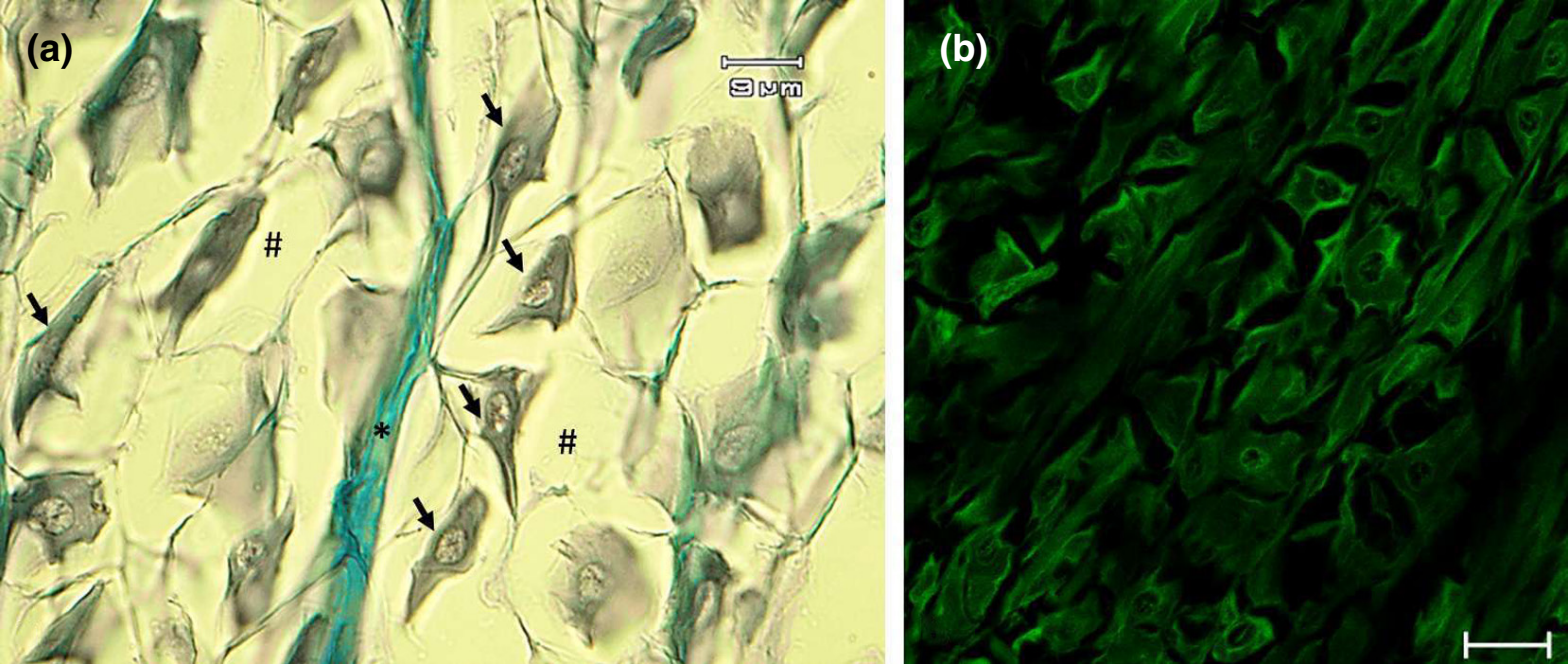
Light micrographs (Masson's trichrome with aniline blue staining) showing (a) the mesenchymal tissue with star‐shaped cells (arrows) immersed in abundant extracellular matrix (hashtag), with an evident septum of connective tissue (asterisk). (b) Vimentin positive star‐shaped cells. Scale bar: a: 9 μm; b: 20 μm

**FIGURE 7:**
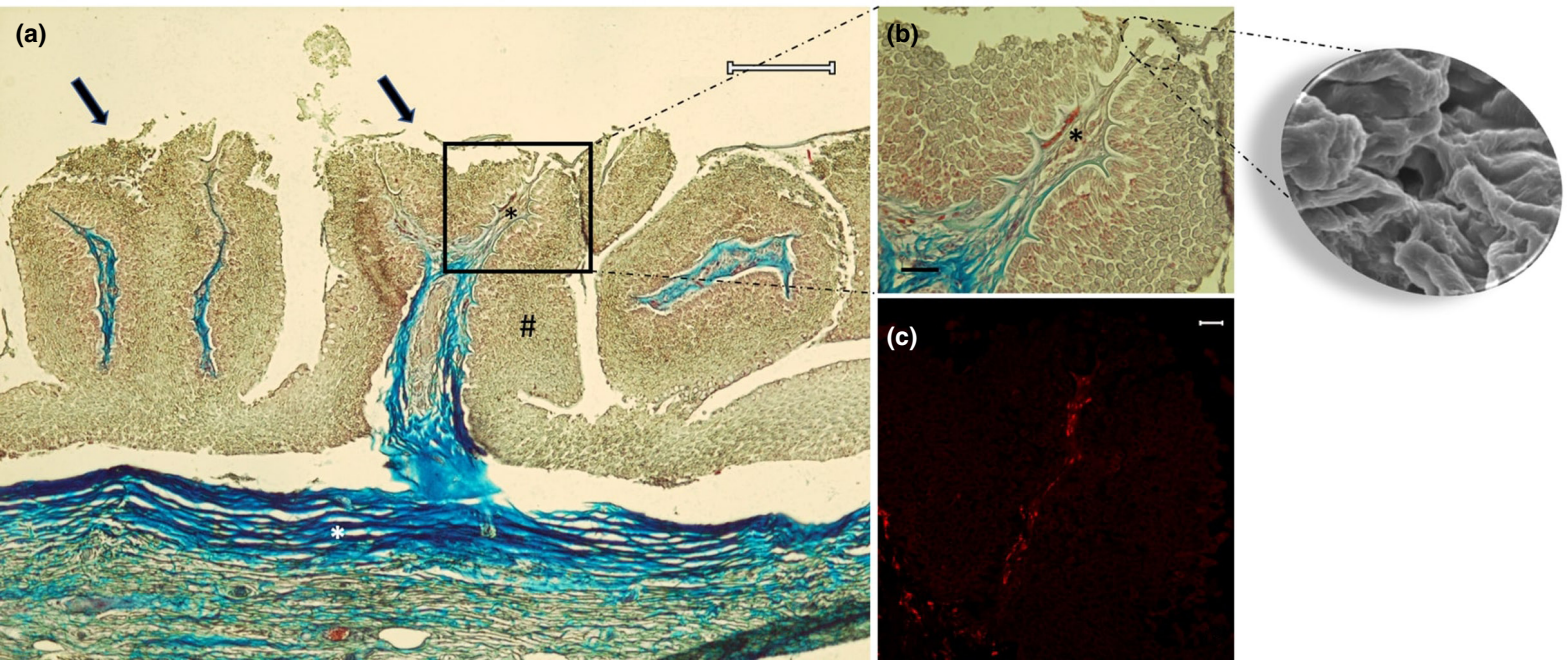
Light micrographs (Masson's trichrome with aniline blue staining) showing (a) in the body and root fungiform‐like papillae (arrows). The mucosa (hashtag) raises in plicae showing the papillae. The connective tissue (white asterisk) throws itself in the mucosa bringing vessels and nerves (black asterisk). (b) A taste pore showed also in the scanning electron micrograph insert, and vessels and nerve (asterisk). (c) Note the presence of papillae with S100 positive nervous fibres. Scale bar: a: 200 μm; b: 30 μm; c: 20 μm

**FIGURE 8:**
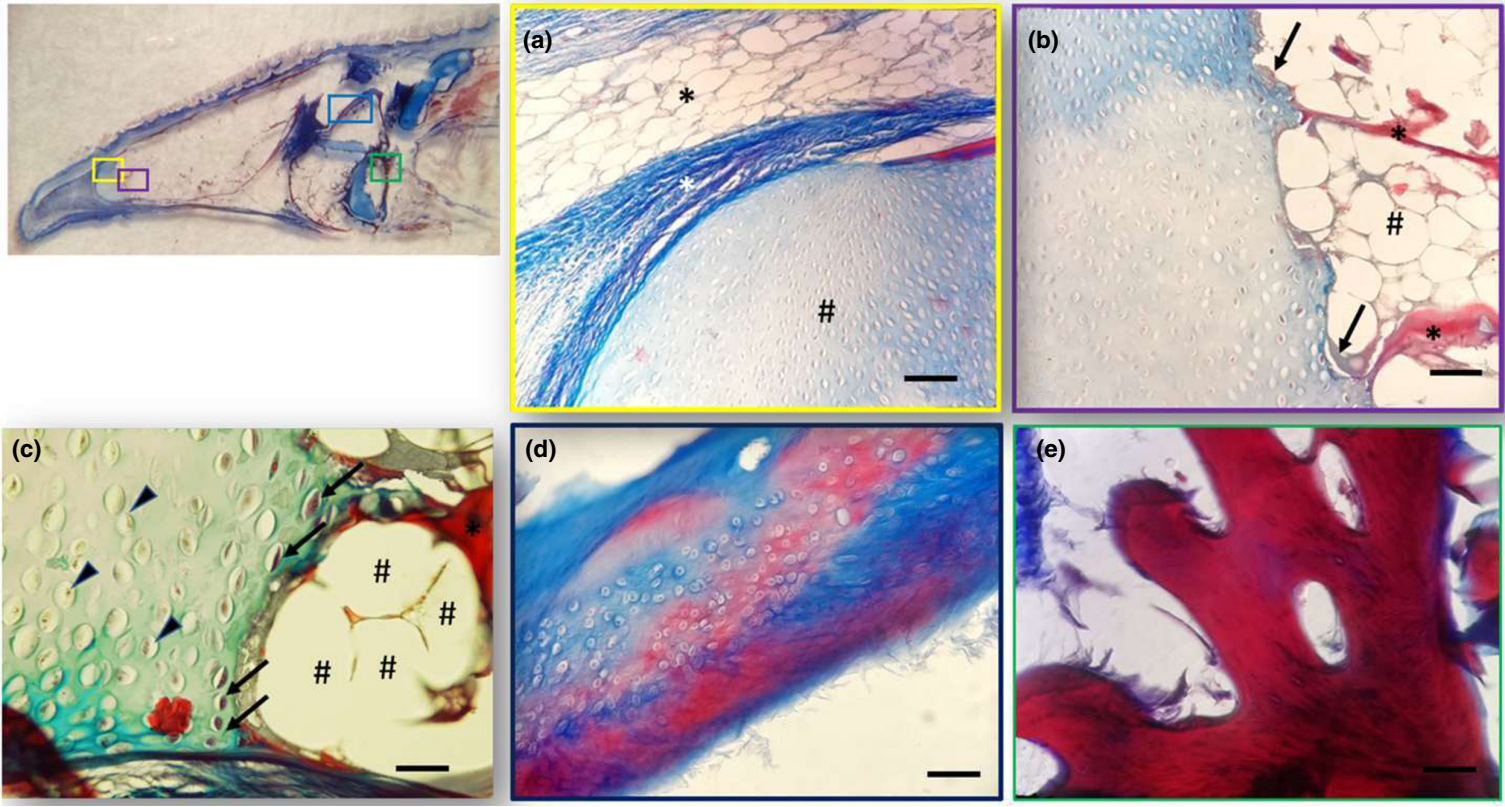
Light micrographs (Masson's trichrome with aniline blue staining) showing (a) different areas of the tongue: (a) Between the apex and the body the unilocular adipose tissue (black asterisk), the dense connective tissue (white asterisk) and the hyaline cartilage pad (hashtag). (b) Hyaline cartilagineous tissue (×) and an ossification process of the cartilage (arrows). A bone trabecula is indicated by the asterisk with a close adipose tissue (hashtags). (c) Chondrocytes (arrowheads) and degenerated chondrocytes (arrows). The bone tissue (asterisk) with a close adipose tissue (hashtags). (d) Hyaline cartilage is stained with blue and cartilage in red during the process of calcification. (e) Bone trabecula at higher magnification. Scale bar: a, b, d: 20 μm; c, e: 30 μm

The dorsal surface of the tongue was covered by a weakly keratinized stratified squamous epithelium. In the apex, body and root, profiles of fungiform‐like papillae projected among the epithelial laminae (Figure 5a). In the tongue, the weakly keratinized epithelium was followed by layers of dense fibrillar connective tissue that evolved into loose connective tissue with interspersed amorphous substance. This layer was formed by unilocular adipose tissue formed by groups of polyhedric fat cells (Figure 5b). At the apex, below the adipose tissue, the connective tissue deepens to delineate lodges within an amorphous extracellular matrix, containing vimentin positive star‐shaped cells immersed in abundant extracellular matrix, thus forming a mesenchymal tissue (Figures 5c, 6a, b). In the body and root, there were folds of mucosa with abundant fungiform‐like papillae (Figure 7a) and presence of taste pores (Figure 7b). Within the papillae, S100 positive nervous fibres were observed (Figure 7c). In the deeper layers of the body, the dense connective tissue showed a pad of hyaline cartilage, with numerous chondrocytes immersed in an amorphous substance (Figure 8a). The posterior margin of this cartilaginous tissue showed an indirect ossification with the formation of trabeculae, aborally projecting through the whole body to show areas filled with adipose tissue (Figure 8b). In the deepest layer, the pre‐existing cartilaginous tissue begins to be ossified forming niches containing adipose tissue (Figure 8c–e).

There were no remarkable differences between the male and female specimens used in this study.

## 4 DISCUSSION

The aim of our study was to analyse the morphological characteristic of the tongue to demonstrate the possible correlations to the mechanisms of prehension and ingestion of food, by scanning electron, light and confocal laser microscopy. So far, the numerous data available regarding the morphology of the oral cavity of fishes (for a review see Kapoor & Khanna, 1994) and the close interrelationships between the oral cavity and feeding habits, and different modes of deglutition have been clearly demonstrated. In addition, the taste system is affected by environmental changes caused by pollution and contaminant (Kasumyan, 2019). The blackspot seabream was chosen in this study because it may become a more widely bred species soon. Prehension and swallowing, in this species, is achieved by the presence of cardiform teeth whose orientation gives important support to ingestion, and their activity is strongly supported by rows of molariform teeth. Several fungiform‐like papillae, through the tongue dorsal surface, whereas no taste buds, were observed. The absence of taste buds has been previously demonstrated in the swordfish and Atlantic salmon tongue, but unlike these two species, in the blackspot seabream the presence of several taste pores was observed, with, under the papillae, S100 positive nervous fibres, thus demonstrating, a taste capacity that should be considered, in aquaculture, in the possible food choices (Benetti et al., 2009; Igbokwe et al., 2021). The presence of folds, several papillae, teeth with different appearance and taste pores could also be compared with previous data obtained, using the same techniques, in gilthead seabass (*Dicentrarchus labrax*) and seabream (*Sparus aurata*), two species widely reared in different countries, especially in the Mediterranean area, in which, as in the blackspot seabream an apex, a body and a root, can be distinguished on the tongue dorsal surface. In the sea bass, numerous canine‐like tooth pads were present along the dorsal surface and the observation of numerous taste buds in different areas of the tongue surface relevant; whereas in the seabream, the apical part of the tongue is inserted into a pouch with a characteristic medial ridge on the body surface and numerous taste buds were scattered all over the dorsal surface. The morphological characteristics of the tongue are strictly related to the environment they live, the evolutionary changes related to it, and most of all, to the diet, whereas no connection is supposed with the age and the weight of the fish. Additionally, in the apex, we observed the presence, below the adipose tissue, of areas in which vimentin positive star‐shaped cells were evident, as mesenchymal cells. Cell typing is confirmed by the clear immunoreactivity for vimentin, and although vimentin is not a specific marker, it is widely used as a marker of mesenchymal‐derived cells as their main cytoskeletal component (Musaelyan et al., 2018). The mesenchymal/stem stromal cells can be found in adult mesenchymal tissues other than bone marrow (Bernardo et al., 2009; Campagnoli et al., 2001) and mesenchyme, as a matrix with morphogenetic properties for several resulting tissues, differs in adipose tissue from spindle mesenchymal cells and, in particular, adipocytes are derived from undifferentiated mesenchymal lineage cells. Fish may retain the ability to regenerate mesenchymal cells as occurs in other structures such as sensory cells (Germanà et al., 2009; Montalbano et al., 2018b). These results confirm our previous data regarding the presence of niches of mesenchymal stem cells niches, in the tongue of Atlantic salmon and rainbow trout (Abbate et al., 2020a, b). The presence and the characteristics of the mesenchymal stem cells, demonstrated in this study, should be further analysed by other different techniques. The demonstration of immunoreactivity of vimentin in fish tongue could be applied in diagnostic purposes of fish pathologies as has been done in tissue and organs of several mammals. Moreover, our results demonstrate that the presence of teeth on both jaws and of taste pores on the tongue dorsal surface is strictly related to the feeding habits of this fish carnivorous species. The pointed conical teeth, with aborally oriented apex, together with the innermost band of cardiform teeth, play a fundamental role in holding the prey and preventing it from escaping during capture. At the same time, the presence of molariform teeth, suitable for crushing, arranged in several rows, suggests that *P*. *bogaraveo* can also feed on bivalve molluscs and crustaceans. In addition, the presence of taste pore on the dorsal surface of the tongue indicates a gustatory function and therefore selective perception in this species.

## 5 CONCLUSION

Considering that there are limited data so far available on the morphological characteristics of the tongue in the blackspot seabream, the current results could contribute to the anatomical knowledge of the digestive system in fish, giving a support for the future wider use of this fish as a farmed species in aquaculture. The morphological data regarding the tongue structure could be utilized as basis for further physiological and nutritional studies aimed at optimization of the blackspot seabream farming, using well‐targeted nutritional protocols. It is known that a better nutrition reflects in better fish welfare and therefore optimum production for human nutrition.

## CONFLICT OF INTEREST

The authors declare that they have no conflict of interest.

## Data Availability

The data that support the findings of this study are available from the corresponding author upon reasonable request.

## References

[ahe12769-bib-0001] Abbate, F. , Germana, G. P , De Carlos, F. , Montalbano, G. , Laura, R. , Levanti, M. B , & Germana, A. (2006). The oral cavity of the adult zebrafish (Danio rerio). Anatomia Histologia Embryologia, 35, 299–304. 10.1111/j.1439-0264.2006.00682.x 16968248

[ahe12769-bib-0002] Abbate, F. , Guerrera, M. C. , Cavallaro, M. , Montalbano, G. , Germanà, A. , & Levanti, M. (2017). LM and SEM study on the swordfish (Xiphias gladius) tongue. Tissue and Cell, 49(6), 633–637. 10.1016/j.tice.2017.09.007 29042066

[ahe12769-bib-0003] Abbate, F. , Guerrera, M. C. , Levanti, M. , Laurà, R. , Aragona, M. , Mhalhel, K. , Montalbano, G. , & Germanà, A. (2020b) Anatomical, histological and immunohistochemical study of the tongue in the rainbow trout (Oncorhynchus mykiss). Anatomia Histologia Embryologia, 49(6), 848–858. 10.1111/ahe.12593 32705711

[ahe12769-bib-0004] Abbate, F. , Guerrera, M. C. , Levanti, M. , Laurà, R. , Montalbano, G. , Cavallaro, M. , & Germanà, A. (2020a). Morphology of the Atlantic salmon (Salmo salar) tongue. Anatomia Histologia Embryologia, 49(6):686–694. 10.1111/AHE.12563 32378253

[ahe12769-bib-0005] Abbate, F. , Guerrera, M. C. , Levanti, M. , Laurà, R. , Montalbano, G. , Cavallaro, M. , & Germanà, A. (2020c). The tongue of Leopard Gecko (Eublepharis macularius). Anatomia Histologia Embryologia, 49, 51–59. 31512785 10.1111/ahe.12483

[ahe12769-bib-0006] Abbate, F. , Guerrera, M. C. , Montalbano, G. , Ciriaco, E. , & Germanà, A. (2012b). Morphology of the tongue dorsal surface of gilthead seabream (Sparus aurata). Microscopy Research and Technique, 75, 1666–1671. 22965546 10.1002/jemt.22114

[ahe12769-bib-0007] Abbate, F. , Guerrera, M. C. , Montalbano, G. , De Carlos, F. , Suarez, A. A. , Ciriaco, E. , & Germanà, A. (2012a). Morphology of the European sea bass (Dicentrarchus labrax) tongue. Microscopy Research and Technique, 75, 643–649. 10.1002/jemt.21105 22505185

[ahe12769-bib-0008] Abbate, F. , Guerrera, M. C. , Montalbano, G. , Zichichi, R. , Germanà, A. , & Ciriaco, E. (2010). Morphology of the lingual dorsal surface and oral taste buds in Italian lizard (Podarcis sicula). Anatomia Histologia Embryologia, 39, 167–171. 20377553 10.1111/j.1439-0264.2010.00992.x

[ahe12769-bib-0009] Abbate, F. , Latella, G. , Montalbano, G. , Guerrera, M. C. , Germanà, G. P. , & Levanti, M. B. (2009). The lingual dorsal surface of the bluetongue skink (Tiliqua scincoides). Anatomia Histologia Embryologia, 38, 348–350. 19769569 10.1111/j.1439-0264.2009.00952.x

[ahe12769-bib-0010] Abbate, F. , Latella, G. , Montalbano, G. , Guerrera, M. C. , Levanti, M. B. , & Ciriaco, E. (2008). Scanning electron microscopical study of the lingual epithelium of green iguana (Iguana iguana). Anatomia Histologia Embryologia, 37, 314–316. 18279492 10.1111/j.1439-0264.2008.00847.x

[ahe12769-bib-0011] Amato, V. , Viña, E. , Calavia, M. G. , Guerrera, M. C. , Laurà, R. , Navarro, M. , De Carlos, F. , Cobo, J. , Germanà, A. , & Vega, J. A. (2012). TRPV4 in the sensory organs of adult zebrafish. Microscopy Research and Technique, 75, 89–96. 21678526 10.1002/jemt.21029

[ahe12769-bib-0012] Audira, G. , Sarasamma, S. , Chen, J. R. , Juniardi, S. , Sampurna, B. P. , Liang, S. T. , Lai, Y. H. , Lin, G. M. , Hsieh, M. C. , & Hsiao, C. D. (2018). Zebrafish mutants carrying leptin a (Lepa) gene deficiency display obesity, anxiety, less aggression and fear, and circadiarhythm and color preference dysregulation. International Journal of Molecular Science, 19(12), 4038. 10.3390/ijms19103195 PMC6320766 30551684

[ahe12769-bib-0013] Bels, V. L. , Jamniczky, H. A. , Montuelle, S. , Pallandre, J. P. , Kardong, K. V. , & Russell, A. P. (2020). Mechanics and kinematics of fluid uptake and intraoral transport in the leopard gecko (Gekkota: Eublepharidae: Eublepharis macularius). Journal of Zoology, 311, 33–34. 10.1111/jzo.12763

[ahe12769-bib-0014] Benetti, E. J. , Pícoli, L. C. , Guimarães, J. P. , Motoyama, A. A. , Miglino, M. A. , & Watanabe, L. S. (2009). Characteristics of filiform, fungiform and vallate papillae and surface of interface epithelium‐connective tissue of the maned sloth tongue mucosa (Bradypus torquatus, Iliger, 1811): Light and scanning electron microscopy study. Anatomia Histologia Embryologia, 38, 42–48. 19143682 10.1111/j.1439-0264.2008.00890.x

[ahe12769-bib-0015] Bernardo, M. E. , Avanzini, M. A. , Ciccocioppo, R. , Perotti, C. , Corneta, A. M. , Moretta, A. , & Locatelli, F. (2009). Phenotypical/functional characterization of in vitro expanded mesenchymal stromal cells from patients with Crohn's disease. Cytotherapy, 11(7), 825–836. 10.3109/14653240903121260 19903096

[ahe12769-bib-0016] Campagnoli, C. , Roberts, I. A. , Kumar, S. , Bennett, P. R. , Bellantuono, I. , & Fisk, N. M. (2001). Identification of mesenchymal stem/progenitor cells in human first‐trimester fetal blood, liver, and bone marrow. Blood, 98, 2396–2402. 10.1182/blood.V98.8.2396 11588036

[ahe12769-bib-0017] Carnovali, M. , Luzi, L. , Terruzzi, I. , Banfi, G. , & Mariotti, M. (2018). Metabolic and bone effects of high‐fat diet in adult zebrafish. Endocrine, 61, 317–326. 10.1007/s12020-017-1494-z 29274064

[ahe12769-bib-0018] Cizek, P. , Hamouzova, P. , Kvapil, P. , & Kyllar, M. (2019). Light and scanning electron microscopy of the tongue of the sand lizard (Lacerta agilis). Folia Morphologica, 78, 101–106. 30009360 10.5603/FM.a2018.0064

[ahe12769-bib-0019] Dos Santos, M. L. , Arantes, F. P. , Santiago, K. B. , & Dos Santos, J. E. (2015). Morphological characteristics of the digestive tract of Schizodon knerii (Steindachner, 1875), (Characiformes: Anostomidae): An anatomical, histological and histochemical study. Anais Da Academia Brasileira De Ciencias, 87, 867–878. 10.1590/0001-3765201520140230 26131636

[ahe12769-bib-0021] Erdogan, S. , & Alan, A. (2012). Gross anatomical and scanning electron microscopic studies of the oropharyngeal cavity in the European magpie (Pica pica) and the common raven (Corvus corax). Microscopy Research and Technique, 75, 379–387. 10.1002/jemt.21067 21898667

[ahe12769-bib-0022] Erdoğan, S. , & Iwasaki, S. (2014). Function‐related morphological characteristics and specialized structures of the avian tongue. Annals of Anatomy, 196, 75–87. 10.1016/j.aanat.2013.09.005 24219998

[ahe12769-bib-0023] Garcia‐Suarez, O. , Cabo, R. , Abbate, F. , Randazzo, B. , Laurà, R. , Piccione, G. , Germanà, A. , & Levanti, M. (2018). Presence and distribution of leptin and its receptor in the gut of adult zebrafish in response to feeding and fasting *Anatomia Histologia* . Embryologia, 47, 456–465. 10.1111/ahe.12384 29998487

[ahe12769-bib-0024] Germanà, A. , Montalbano, G. , Guerrera, M. C. , Laurà, R. , Levanti, M. , Abbate, F. , de Carlos, F. , Vega, J. A. , & Ciriaco, E. (2009). Sox‐2 in taste bud and lateral line system of zebrafish during development. Neuroscience Letters, 467, 36–39. 10.1016/j.neulet.2009.09.056 19800392

[ahe12769-bib-0025] Guerrera, M. C. , Montalbano, G. , Germanà, A. , Maricchiolo, G. , Ciriaco, E. , & Abbate, F. (2015). Morphology of the tongue dorsal surface in white sea bream (Diplodus sargus sargus). Acta Zoologica, 39, 167–171.

[ahe12769-bib-0026] Herrel, A. , Redding, C. L. , Meyers, J. J. , & Nishikawa, K. C. (2014). The scaling of tongue projection in the veiled chameleon, Chamaeleo calyptratus. Zoology (Jena), 117, 227–236. 10.1016/j.zool.2014.01.001 24703241

[ahe12769-bib-0027] Iaconisi, V. , Marono, S. , Parisi, G. , Gasco, L. , Genovese, L. , Maricchiolo, G. , Bovera, F. , & Piccolo, G. (2017). Dietary inclusion of Tenebrio molitor larvae meal: Effects on growth performance and final quality treats of blackspot sea bream (Pagellus bogaraveo). Aquaculture, 476, 49–58. 10.1016/j.aquaculture.2017.04.007

[ahe12769-bib-0028] Igbokwe, C. O. , Bello, U. M. , & Mbajiorgu, F. E. (2021). Anatomical and surface ultrastructural investigation of the tongue in the straw‐coloured fruit bat (Eidolon helvum, Kerr 1972). Anatomia Histologia Embryologia, 50, 448–458. 33350508 10.1111/ahe.12648

[ahe12769-bib-0029] Ikpegbu, E. , Ibe, C. S. , & Nlebedum, U. C. (2019). Digestive tract of the false upside down catfish (Synodontis nigrita) from river Benue Nigeria: A micro‐morphological investigation. Studia Universitatis “vasile Goldiş”. Seria Ştiinţele Vieţii, 29, 53–64.

[ahe12769-bib-0030] Kapoor, B. G. , & Khanna, B. (1994). The alimentary canal of teleosts: A brief survey of structure and function. In H. R. Singh (Ed.), Advances in fish biology (pp. 12–24). Hindus‐tan Publishing Corporation.

[ahe12769-bib-0031] Kasumyan, A. O. (2019). The taste system in fishes and the effects of environmental variables. Journal of Fish Biology, 155–178. 10.1111/jfb.13940 30793305

[ahe12769-bib-0032] Kettratad, J. , Senarat, S. , Boonyoung, P. , & Jiraungkoorsku, W. (2017). Tongue anato histology of the oceanodromous adult Rastrelliger brachysoma (Bleeker, 1851) with a note on the comparison with the tongue structure of adult R. kanagurta (Cuvier, 1816). Songklanakarin Journal of Science Technology, 39, 117–121.

[ahe12769-bib-0033] Levanti, M. , Germanà, A. , Montalbano, G. , Guerrera, M. C. , Cavallaro, M. , & Abbate, F. (2017). The tongue dorsal surface in fish: A comparison among three farmed species. Anatomia Histologia Embryologia, 46, 103–109. 10.1111/ahe.12259 27990675

[ahe12769-bib-0034] Mahmoud, U. M. , Essa, F. , & Sayed, A. E. (2016). Surface architecture of the oropharyngealcavity and the digestive tract of Mulloidichthys flavolineatus from the red sea, Egypt: A scanning electron microscope study. Tissue and Cell, 48, 624–633. 10.1016/j.tice.2016.09.001 27641971

[ahe12769-bib-0035] Mania, M. , Maruccio, L. , Russo, F. , Abbate, F. , Castaldo, L. , D'Angelo, L. , de Girolamo, P. , Guerrera, M. C. , Lucini, C. , Madrigrano, M. , Levanti, M. , & Germanà, A. (2017). Expression and distribution of leptin and its receptors in the digestive tract of DIO (diet‐induced obese) zebrafish. Annals of Anatomy, 212, 37–47. 10.1016/j.aanat.2017.03.005 28477448

[ahe12769-bib-0036] Micale, V. , Genovese, L. , Guerrera, M. C. , Laurà, R. , Maricchiolo, G. , & Muglia, U. (2011). The reproductive biology of *Pagellus bogaraveo,* a new candidate species for aquaculture. The Open Marine Biology Journal, 5, 42–46.

[ahe12769-bib-0037] Modina, S. , Abbate, F. , Germanà, G. P. , Lauria, A. , & Luciano, A. M. (2007). β‐Catenin localization and timing of early development of bovine embryos obtained from oocytes matured in the presence of follicle stimulating hormone. Animal Reproduction Science, 100, 264–279. 10.1016/j.anireprosci.2006.07.008 16956737

[ahe12769-bib-0038] Montalbano, G. , Capillo, G. , Laurà, R. , Abbate, F. , Levanti, M. , Guerrera, M. C. , Ciriaco, E. , & Germanà, A. (2018b). Neuromast hair cells retain the capacity of regeneration during heavy metal exposure. Annals of Anatomy, 218, 183–189. 10.1016/j.aanat.2018.03.007 29719206

[ahe12769-bib-0039] Montalbano, G. , Mania, M. , Abbate, F. , Navarra, M. , Guerrera, M. C , Laura, R. , Vega, J. A , Levanti, M. , & Germanà, A. (2018). Melatonin treatment suppresses appetite genes and improves adipose tissue plasticity in diet‐induced obese zebrafish. Endocrine, 62, 381–393. 10.1007/s12020-018-1653-x 29926348

[ahe12769-bib-0040] Montalbano, G. , Mania, M. , Guerrera, M. C. , Abbate, F. , Laurà, R. , Navarra, M. , & Germanà, A. (2016). Morphological differences in adipose tissue and changes in BDNF/Trkb expression in brain and gut of a diet induced obese zebrafish model. Annals of Anatomy, 204, 36–44. 10.1016/j.aanat.2015.11.003 26617157

[ahe12769-bib-0041] Musaelyan, A. , Lapin, S. , Nazarov, V. , Tkachenko, O. , Gilburd, B. , Mazing, A. , Mikhailova, L. , & Shoenfeld, Y. (2018). Vimentin as antigenic target in autoimmunity: A comprehensive review. Autoimmunity Reviews, 17(9), 926–934. 10.1016/j.autrev.2018.04.004 30009963

[ahe12769-bib-0042] Sadeghinezhad, J. , Rahmati‐Holasoo, H. , Fayyaz, S. , & Zargar, A. (2015). Morphological study of the northern pike (Esox lucius) tongue. Anatomical Science International, 90, 235–239. 10.1007/s12565-014-0254-x 25205560

[ahe12769-bib-0043] Viña, E. , Parisi, V. , Abbate, F. , Cabo, R. , Guerrera, M. C. , Laurà, R. , Quirós, L. M. , Pérez‐Varela, J. C. , Cobo, T. , Germanà, A. , Vega, J. A. , & García‐Suárez, O. (2015). Acid‐sensing ion channel 2 (ASIC2) is selectively localized in the cilia of the non‐sensory olfactory epithelium of adult zebrafish. Histochemistry and Cell Biology, 143, 59–68. 10.1007/s00418-014-1264-4 25161120

